# Antiphospholipid Antibodies in Severe COVID-19: Frequency, Clinical Features, and 12-Week Follow-Up

**DOI:** 10.7759/cureus.100657

**Published:** 2026-01-03

**Authors:** Md. Nazmul Hasan, Fazle R Chowdhury, Kazi Mohammad Kamrul Islam, Chowdhury Adnan Sami, Mohammad Tanvir Islam

**Affiliations:** 1 Internal Medicine, Bangabandhu Sheikh Mujib Medical University, Dhaka, BGD; 2 Hematology, Bangladesh Medical University, Dhaka, BGD; 3 Medicine, Dhaka Community Medical College, Dhaka , BGD

**Keywords:** antiphospholipid antibodies, autoantibodies, bangladesh, covid-19-associated coagulopathy, thrombosis

## Abstract

Background: Severe COVID-19 is characterized by a hypercoagulable state and elevated thrombotic risk, potentially linked to antiphospholipid antibodies (APLAs). The prevalence and clinical significance of APLAs in critically ill patients, particularly in low- and middle-income countries such as Bangladesh, remain unknown.

Methods: We conducted a prospective observational study among 100 adult patients admitted with severe COVID-19 to tertiary hospitals in Bangladesh between June 2021 and February 2022. We measured APLAs (IgM and IgG) and anticardiolipin antibodies (aCL; IgM and IgG) in serum samples obtained within 48 hours of admission. Patients with initially positive antibodies underwent follow-up testing at 12 weeks. We collected demographic, clinical, and laboratory data, along with thromboembolic events. Statistical analysis included paired t-tests and effect size estimation.

Results: Of the 100 patients prospectively enrolled, 57 were men, and the mean age was 60 (± 13.4) years. A total of 30 patients tested positive for at least one antibody at baseline. We observed a major decrease in APLA levels between baseline and week 12. Mean APLA IgM decreased from 13.95±10.53 to 5.12±6.60 (p=0.002) and APLA IgG levels from 18.8±37.42 to 5.64±5.22 (p=0.048). Similarly, aCL IgM declined from 16.68±9.75 to 5.49±6.73 (p<0.001), and aCL IgG dropped from 8.90±8.22 to 4.35±3.66 (p=0.011), both of which were statistically significant. Only two antibody-negative patients presented with acute myocardial infarction.

Conclusion: We detected APLAs in one third of patients with severe COVID-19; however, they were not associated with clinical thromboembolic events and decreased markedly over time. These findings indicate that APLA responses during acute COVID-19 are largely transient and may not be useful for predicting thrombotic risk. Our findings do not support routine APLA testing during the acute phase of COVID-19.

## Introduction

COVID-19, caused by severe acute respiratory syndrome coronavirus 2 (SARS-CoV-2), has triggered an unprecedented pandemic, impacting health care systems worldwide. Although most infections result in mild-to-moderate respiratory illness, a considerable proportion of patients develop severe complications, including acute respiratory distress syndrome (ARDS), thromboembolic disorders, and multiorgan failure [1]. A prominent pathological hallmark of severe COVID-19 is a hypercoagulable state that markedly increases the incidence of thrombotic complications, such as deep vein thrombosis (DVT), pulmonary embolism (PE), and ischemic stroke [2]. The pathophysiology underlying this hypercoagulable state remains incompletely elucidated and is the focus of intensive research, with emerging evidence supporting an autoimmune-mediated etiology, including associations with antiphospholipid antibodies (APLAs) [3] directed against phospholipid-binding proteins, namely lupus anticoagulant (LA), anticardiolipin antibodies (aCL), and anti-beta-2-glycoprotein-I antibodies (anti-β2GPI) [4]. These antibodies represent the laboratory hallmark of antiphospholipid syndrome (APS), an autoimmune disorder characterized by arterial and venous occlusion, miscarriages, and systemic inflammation [5]. Although APLAs are well documented in systemic lupus erythematosus, recent evidence indicates that various infections, including viral infections, can trigger transient or persistent production of these antibodies [6].

Authors of international studies have reported that SARS-CoV-2 infection can trigger APLAs that increase thrombosis risks in critically ill patients, leading to adverse clinical outcomes [7]. These observations suggest that severe COVID-19 may induce an acquired prothrombotic milieu resembling APS and warrant further population-specific investigation. In Bangladesh, clinicians have associated severe COVID-19 with increased thrombotic complications, yet the underlying immunological mechanisms remain poorly understood [8]. Although numerous researchers worldwide have characterized COVID-19-associated coagulopathy, data on the prevalence and clinical significance of APLAs in Bangladeshi patients are lacking. Considering the distinct genetic, environmental, and healthcare-related discrepancies in Bangladesh, evaluating the role of APLAs in severe COVID-19 in this population is essential for effective treatment.

This study aimed primarily to determine the prevalence and temporal behavior of APLAs among adults hospitalized with severe COVID-19 in Bangladesh. We also evaluated the association between APLA positivity and clinical outcomes, particularly thrombotic events, and assessed the persistence or decline of antibody titers at 12-week follow-up. This study aimed to evaluate the clinical significance of infection-induced APLAs in a South Asian population.

## Materials and methods

Study design and setting

We conducted this prospective observational study at Bangabandhu Sheikh Mujib Medical University (BSMMU), a tertiary-care hospital in Bangladesh, from June 2021 to February 2022. The primary objective was to assess the frequency, persistence, and clinical significance of APLAs in adults with severe COVID-19.

Ethical consideration

This study involved human participants, so ethical clearance was taken from the Institutional Review Board (IRB) of BSMMU (BSMMU/2020/10175). We obtained informed written consent from all the participants.

Study population

We enrolled adult patients admitted with severe COVID-19 (age ≥18; n = 100). We defined severe disease according to World Health Organization criteria: presence of ARDS, oxygen saturation <90% on room air, or requirement for high-flow oxygen or mechanical ventilation. Exclusion criteria were a known diagnosis of APS, established autoimmune disease, or regular anticoagulant therapy.

Sample collection and laboratory measurements

We collected venous blood samples (5 mL) from all participants within 48 hours of hospital admission. We separated sera and measured antibodies using standardized enzyme-linked immunosorbent assay (ELISA) kits. APLAs (IgG/IgM) and anticardiolipin antibodies (aCL IgG/IgM) were measured using an ELISA-based assay on an ELISA Plate Reader (DAS, Italy) at the Department of Microbiology, BSMMU. Results were expressed in U/mL, and interpretation followed the laboratory’s validated reference ranges: <10 U/mL = negative, 10-15 U/mL = borderline, and >15 U/mL = positive. All tests were performed using the same assay platform throughout the study period to ensure methodological consistency, and internal quality controls were run with each batch. Standard ELISA-based assays were used because they represent the most widely available and validated method for APLA testing in our clinical setting and were feasible to perform consistently during the pandemic. Advanced multiplex platforms and antigen-specific assays (e.g., domain-I β2GPI or multiplex immunoassays) were not available due to resource and supply-chain constraints at the time of the study. Testing included anticardiolipin (aCL) antibodies (IgG, IgM) and APLA antibodies (IgG, IgM). We did not perform LA testing due to logistical constraints.

Collection of clinical and laboratory data

We recorded demographic characteristics, comorbidities (diabetes, hypertension, ischemic heart disease), and clinical presentation using a standardized case report form. Routine laboratory investigations included complete blood count, liver and renal function panels, and inflammatory and coagulation markers; C-reactive protein (CRP), D-dimer, erythrocyte sedimentation rate (ESR), serum ferritin, and lactate dehydrogenase (LDH).

Thrombotic surveillance

Thrombotic events were monitored clinically throughout hospitalization including myocardial infarction (MI), DVT, PE, and ischemic stroke. Diagnostic imaging, including Doppler ultrasonography for suspected DVT, CT pulmonary angiography for suspected pulmonary embolism, and CT/MRI brain for suspected cerebrovascular events was intended to be performed only when clinically indicated, rather than as routine screening. 

Anticoagulation and clinical management

All enrolled patients received prophylactic anticoagulation as per national COVID-19 guidelines, typically with low-molecular-weight heparin (enoxaparin 40 mg once daily), unless contraindicated. Dose escalation (e.g., enoxaparin 40 mg twice daily) was considered in patients when indicated clinically. The specific regimen for each patient was recorded in the case file.

Follow-up evaluation

We reevaluated patients with positive APLAs using the same ELISA modality for APLAs 12 weeks after hospital discharge to assess persistence or resolution of antibody positivity.

Statistical analysis

We expressed continuous variables as mean ± standard deviation (SD) or median (interquartile range), as appropriate. We reported categorical variables as frequencies and percentages. We analyzed differences between baseline and follow-up antibody titers using paired t-tests. We estimated effect sizes using Cohen’s d and Hedges’ g. We considered a p-value < .05 statistically significant. We performed all analyses with IBM SPSS Statistics for Windows, Version 25 (Released 2017; IBM Corp., Armonk, New York, United States).

## Results

Baseline demographic and clinical characteristics

Among the 100 patients with severe disease enrolled, 57 (57%) were men and 43 (43%) were women, and the mean age was 60 (± 13.4) years. There were 53 (53%) aged >61 years, 17 (17%) aged 51-60 years, 20 (20%) aged 41-50 years, and only 10 (10%) were ≤40 years. In the body mass index (BMI) assessment, 54 (54%) were normal weight, 34 (34%) were overweight, and 12 (12%) were obese (Table 1).

**Table 1:** Demographic characteristics of subjects BMI: Body mass index

Characteristics	Number (n)	Percent (%)
Age (years)		
18–40	10	10.0
41–50	20	20.0
51–60	17	17.0
>61	53	53.0
Sex		
Male	57	57.0
Female	43	43.0
BMI		
Healthy	54	54.0
Overweight	34	34.0
Obesity	12	12.0

The cardinal symptoms were dyspnea in 93 patients (93%), fever in 88 patients (88%), and cough in 84 patients (84%). Forty-five (45%) patients had anorexia, 44 (44%) patients had fatigue, and 42 (42%) patients had myalgia as well. Gastrointestinal (GI) and neuro-olfactory symptoms were less common, including diarrhea (8%), nausea (8%), abdominal pain (4%), rash (2%), and eye congestion (2%).

**Figure 1:**
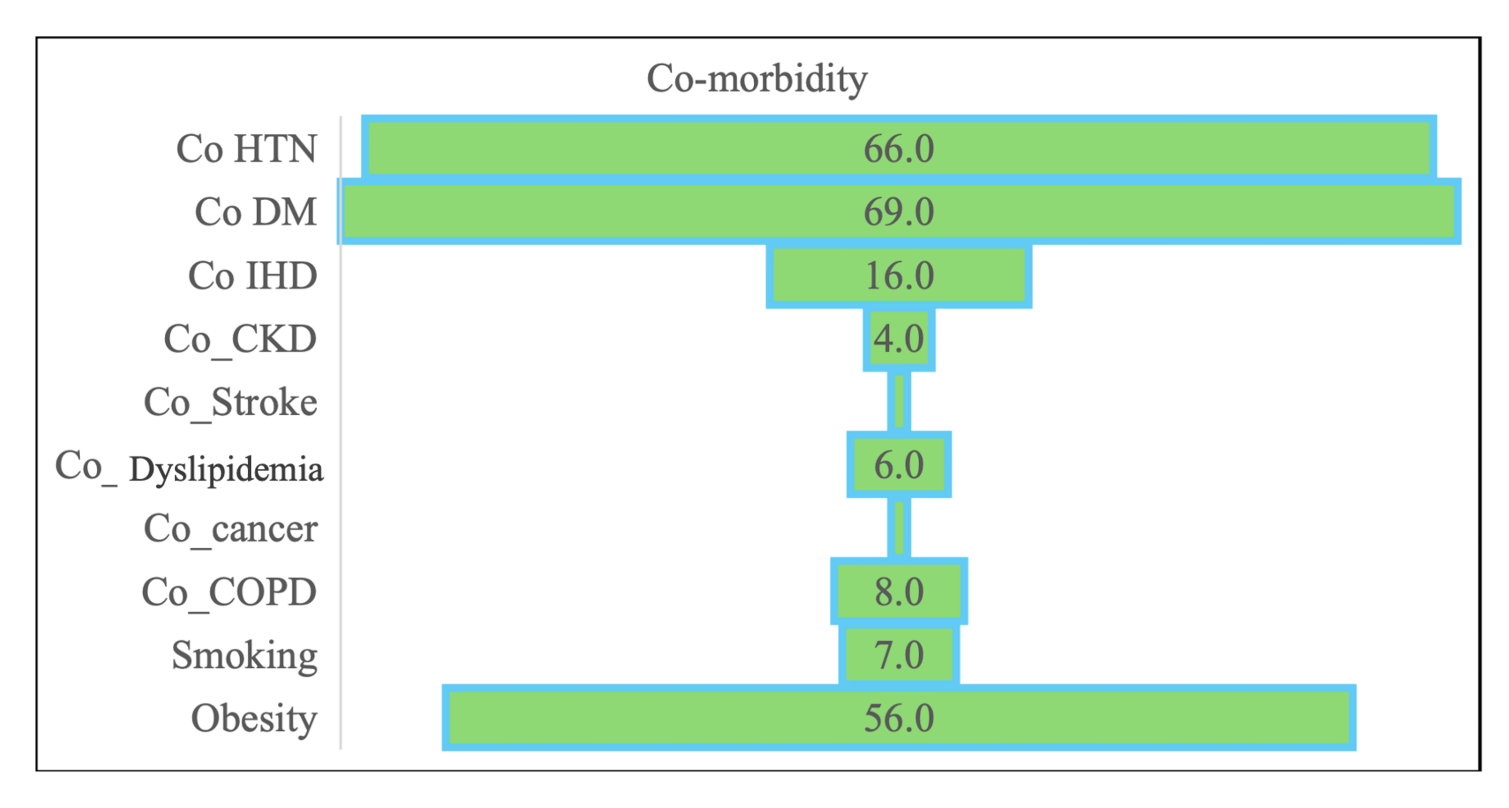
Comorbidity of COVID-19 patients CKD: Chronic kidney disease; COPD: chronic obstructive pulmonary disease; DM: diabetes mellitus; IHD: ischemic heart disease; HTN: hypertension

There was a high proportion of comorbidities: diabetes mellitus in 69 patients (69%), hypertension in 66 patients (66%), ischemic heart disease in 16 patients (16%), chronic obstructive pulmonary disease in eight patients (8%), and chronic kidney disease in four patients (4%). There were 14 (14%) in-hospital deaths, and 86 (86%) patients were discharged after recovery (Figure 1).

APLA positivity at baseline

At baseline, 30 patients were positive for at least one antibody. The mean APLA IgM level was 13.95±10.53 and the mean APLA IgG level was 18.8±37.42 at baseline. Moreover, aCL IgM was 16.68±9.75, and aCL IgG was 8.90±8.22 (Table 2).

**Table 2:** Descriptive statistics of coagulation and antiphospholipid antibody parameters APLA: Antiphospholipid; aCL: anti-cardiolipin antibody; PT: prothrombin time; APTT: activated partial thromboplastin time

Variable	N	Minimum	Maximum	Mean	SE	SD
PT (seconds)	100	11.70	18.90	13.31	0.13364	1.33641
APTT (seconds)	100	25.00	61.80	32.19	0.47311	4.73106
Fibrinogen (mg/dL)	98	128.00	803.30	394.34	15.30968	151.55810
APLA IgM (0 weeks)	100	2.10	32.80	13.95	0.79198	10.53
APLA IgM (12 weeks)	30	2.30	30.20	5.12	1.17033	6.60
APLA IgG (0 weeks)	98	2.44	210.00	18.80	2.18347	37.42
APLA IgG (12 weeks)	30	3.15	22.30	5.64	0.92609	5.22
aCL IgM (0 weeks)	100	2.10	30.20	16.68	0.81450	9.75
aCL IgM (12 weeks)	30	2.02	28.20	5.49	1.19343	6.73
aCL IgG (0 weeks)	98	2.40	30.80	8.90	0.54932	8.22
aCL IgG (12 weeks)	30	2.19	22.80	4.35	0.64717	3.66

Laboratory inflammation profile

We show the laboratory parameters in Table 3. The mean total count was 10.44±5.35×10⁹/L, neutrophils 79.0±12.8%, lymphocytes 16.6±11.8%, and platelets 253.2±120.1×10⁹/L. Inflammatory markers were increased: ESR 52.3±28.5 mm/hr, CRP 66.7 ±60.4mg/L, ferritin 786.7±889.7ng/mL, and D-dimer 0.96±1.61mg/L. Alanine aminotransferase and LDH were also raised; mean creatinine was within the normal range. 

**Table 3:** Descriptive statistics of laboratory parameters CBC: Complete blood count; ESR: erythrocyte sedimentation rate (millimetres per hour); SGPT: serum glutamic pyruvic transaminase; CRP: C-reactive protein; LDH: lactate dehydrogenase

Variable	N	Minimum	Maximum	Mean	SE	SD
CBC – Total Count (×10⁹/L)	99	3.06	32.00	10.44	0.53805	5.35357
CBC – Neutrophils (%)	99	42.00	96.00	79.00	1.29083	12.84364
CBC – Lymphocytes (%)	99	2.00	54.00	16.56	1.18790	11.81941
CBC – Platelets (×10⁹/L)	98	2.00	865.00	253.19	12.13616	120.14190
ESR (mm/hr)	100	0.00	120.00	52.33	2.85233	28.52327
SGPT (ALT, U/L)	99	5.56	320.00	59.27	5.55357	55.25730
CRP (mg/L)	99	0.86	266.20	66.65	6.06985	60.39421
Ferritin (ng/mL)	99	12.50	6465.44	786.74	89.41868	889.70465
D-dimer (mg/L FEU)	100	0.10	9.93	0.9634	0.16067	1.60671
LDH (U/L)	96	29.00	1400.00	370.03	21.96197	215.18249
Creatinine (mg/dL)	98	0.40	7.10	1.14	0.07731	0.76530

Antibody presence at 12 weeks

**Figure 2:**
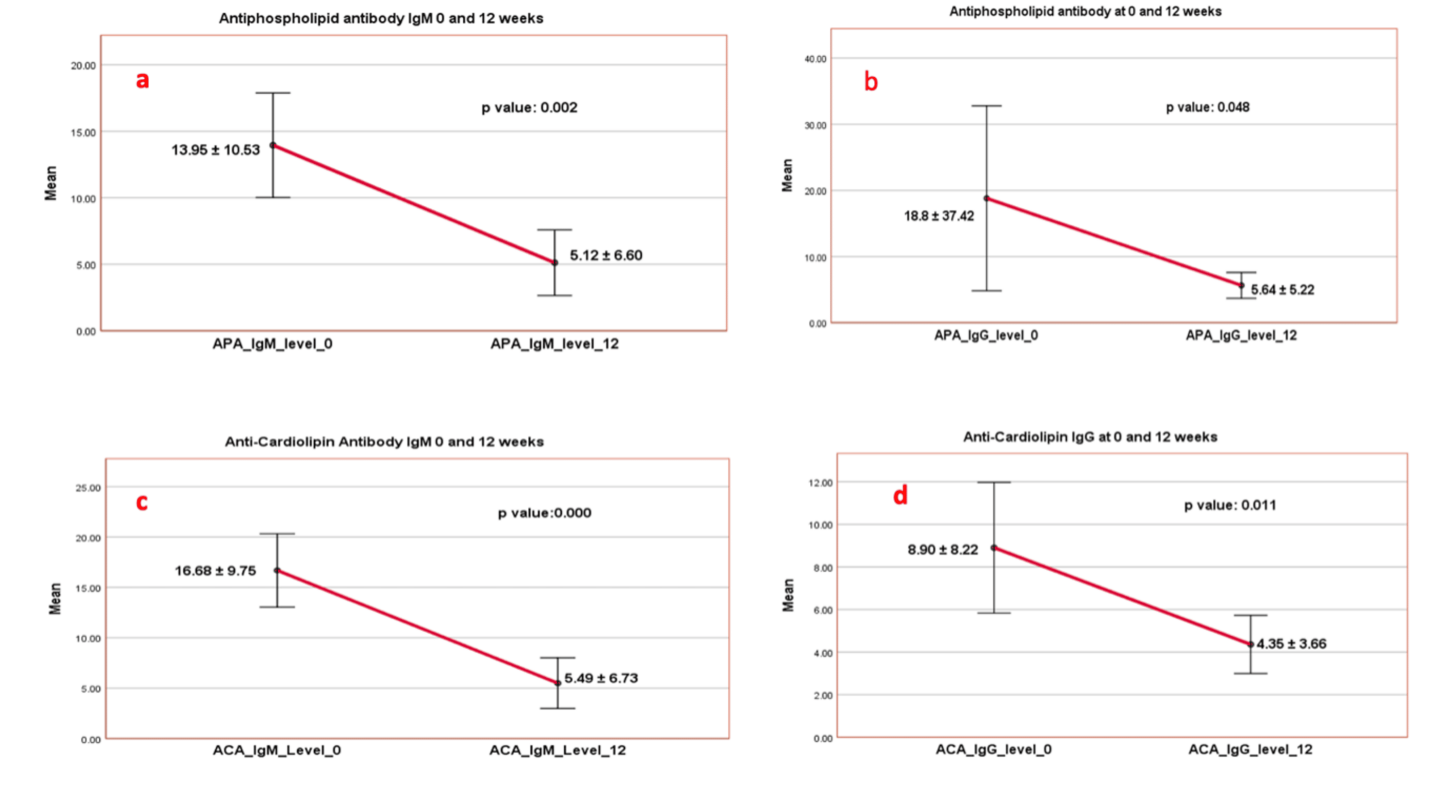
Comparison of initial positive antibody (n=30) at baseline and 12-week level: APLA IgM (a), IgG (b); aCL IgM(c), and IgG (d) APLA: Antiphospholipid; aCL: anti-cardiolipin antibody

Among patients who underwent repeat testing at 12 weeks (30 patients), APLA levels had declined significantly. Mean values at follow-up were as follows: APLA IgM 5.12±6.60, APLA IgG 5.64±5.22, aCL IgM 5.49±6.73, and aCL IgG 4.35±3.66. These values are significantly lower than those recorded at baseline, suggesting substantial temporal decreases in antibody titers (Table 2). APLA levels decreased significantly at baseline and 12 weeks. Mean APLA IgM decreased from 13.95±10.53 to 5.12±6.60 (p=0.002) and APLA-IgG from 18.8±37.42 to 5.64±5.22 (p=0.048). Similarly, aCL IgM declined from 16.68±9.75 to 5.49±6.73 (p<0.001) and aCL IgG from 8.90±8.22 to 4.35±3.66 (p=0.011). The reduction in both isotypes was statistically significant (Figure 2).

Thrombotic phenomena and relationship with antibody

Thrombotic events were rare. No patients developed DVT, PE, or ischemic stroke. Acute MI occurred in only two patients (2%), both of whom were antibody-negative at baseline. Spearman’s correlation analysis revealed no significant association between antibody titers and markers of disease severity or thrombosis (Table 4).

**Table 4:** Spearman correlation of antibody levels with severity markers and laboratory parameters PT: Prothrombin time; APTT: activated partial thromboplastin time; CRP: C-reactive protein; LDH: lactate dehydrogenase

Variable	APLA IgM r	APLA IgM p	APLA IgG r	APLA IgG p	aCL IgM r	aCL IgM p	aCL IgG r	aCL IgG p
Platelet count	0.12	0.906	0.019	0.853	-0.031	0.762	0.147	0.153
CRP	0.14	0.166	0.025	0.808	0.128	0.205	0.083	0.419
Ferritin	-0.049	0.633	-0.037	0.722	0.088	0.387	-0.004	0.972
D-dimer	-0.58	0.567	0.095	0.350	-0.097	0.338	0.140	0.169
PT	0.037	0.715	0.025	0.803	-0.006	0.955	-0.048	0.638
APTT	-0.02	0.844	-0.019	0.856	0.016	0.874	-0.108	0.292
Fibrinogen	0.082	0.420	0.100	0.331	0.036	0.724	0.107	0.300
LDH	-0.122	0.236	-0.090	0.388	-0.044	0.667	0.126	0.227

Interpretation of effect size

To aid interpretation, we summarized effect sizes for key variables rather than focusing on detailed statistical parameters. Age (d=4.47) and BMI (d=5.66) showed very large effect sizes, reflecting the high-risk clinical profile of the study population (Table 5). At baseline, effect sizes for aCL IgM, aCL IgG, and APLA IgM were in the moderate-to-large range, while APLA IgG demonstrated a smaller effect. By 12 weeks, most antibody-related effect sizes decreased, although aCL IgG remained relatively large, suggesting that IgG isotypes may persist longer than IgM. Overall, the effect-size analysis supports our primary observation that APLA titers decline substantially over time, although some immunologic activity may continue in a subset of patients.

**Table 5:** Association between sociodemographic characteristics and antiphospholipid antibody positivity CI: Confidence interval; APLA: antiphospholipid; aCL: anti-cardiolipin antibody; BMI: body mass index

Variable	Effect Size	Standardizer	Point Estimate	95% CI Lower	95% CI Upper
Age	Cohen’s d	13.410	4.471	3.818	5.122
Hedges’ correction	13.513	4.437	3.789	5.083	
BMI	Cohen’s d	4.49800	5.664	4.851	6.475
Hedges’ correction	4.53244	5.621	4.814	6.426	
APLA IgM (0 weeks)	Cohen’s d	7.91977	0.939	0.702	1.173
Hedges’ correction	7.98041	0.932	0.697	1.164	
APLA IgM (12 weeks)	Cohen’s d	6.51611	0.772	0.365	1.169
Hedges’ correction	6.68488	0.752	0.355	1.139	
APLA IgG (0 weeks)	Cohen’s d	1.61528	0.403	0.196	0.608
Hedges’ correction	1.78422	0.400	0.194	0.603	
APLA IgG (12 weeks)	Cohen’s d	5.15623	1.078	0.628	1.517
Hedges’ correction	5.28977	1.051	0.612	1.479	
aCL IgM (0 weeks)	Cohen’s d	8.14503	1.041	0.796	1.283
Hedges’ correction	8.20740	1.033	0.790	1.273	
aCL IgM (12 weeks)	Cohen’s d	6.64475	0.811	0.399	1.213
Hedges’ correction	6.81865	0.790	0.389	1.182	
aCL IgG (0 weeks)	Cohen’s d	5.43800	1.107	0.854	1.357
Hedges’ correction	5.48051	1.099	0.847	1.347	
aCL IgG (12 weeks)	Cohen’s d	3.60329	1.199	0.729	1.657
Hedges’ correction	3.69662	1.169	0.711	1.615	

## Discussion

In this study, we detected APLAs in 30% of patients with severe COVID-19, a prevalence broadly consistent with international reports. Although APLA positivity was relatively common, overt thrombotic events were rare: only two patients experienced MI (2%), and we recorded no cases of venous thrombosis, pulmonary embolism, or ischemic stroke. Importantly, antibody titers declined significantly by 12 weeks, suggesting that APLA responses during acute COVID-19 were predominantly transient.

These findings align with emerging evidence that SARS-CoV-2 infection can induce transient autoantibody production as part of the acute-phase inflammatory response. Early case series raised concerns about COVID-19-associated APS, including the report by Zhang et al. on ischemic strokes in patients with positive anticardiolipin and anti-β2GPI antibodies [4]. Larger subsequent studies, however, revealed considerable variability in APLA positivity. Zuo et al. found detectable APLAs in more than half of hospitalized patients, some of which promoted neutrophil extracellular trap formation in vitro [3]. In a systematic review encompassing more than 14,000 patients, Sabaghian et al. reported APLA positivity in approximately 50% of the participants [9].

LA prevalence in severe COVID-19 has ranged from 35% to 90% [10,11], whereas anticardiolipin and anti-β2GPI antibodies are typically less prevalent and usually present at low titers [3]. Our 30% positivity rate therefore falls within the published range. The paucity of clinical thrombosis despite this frequency suggests that infection-induced APLAs were largely nonpathogenic or were effectively mitigated by routine thromboprophylaxis. Similar findings have emerged from other cohorts; Devreese et al. observed that, even among critically ill patients with frequent LA positivity, no specific APLA profiles, including triple positivity, predicted thrombotic complications [12]. 

Aligning with this, authors of a propensity-matched study in Japan did not observe a difference in APLA prevalence between COVID-19 patients with and without thrombosis (41.9% vs 38.7%), suggesting that the presence of APLAs alone did not increase thrombotic risk [13]. In our cohort, 30% patients had APLAs; however, only 2% developed an arterial thrombotic event and there were no venous thromboses, indicating that APLAs are more often an epiphenomenon of severe infection rather than a direct cause of macrothrombosis [14].

Follow-up data from our study and others support the transient nature of these antibodies. Vollmer et al. showed that LA became negative in most patients upon retesting after several weeks, with no new thrombotic or autoimmune manifestations during follow-up [14]. Similarly, Arcani et al. reported that although up to 30% of survivors remained APLA-positive at 12 weeks, newly diagnosed APS was rare (1.6%), and persistent high-titer profiles consistent with true APS were exceptional [15]. Thus, the majority of APLA responses in COVID-19 appear to represent short-lived, inflammation-driven autoantibodies rather than markers of sustained autoimmune pathology [15,16].

Current expert guidance reflects these observations. Professional societies, including the International Society on Thrombosis and Haemostasis and the American Society of Hematology, recommend against routine APLA testing in COVID-19 because transient antibody elevations are common but rarely influence management or fulfil APS criteria [17]. Our cohort, characterized by high prevalence of metabolic comorbidities and older age, may have shown stronger inflammatory and autoimmune responses; however, the lack of associated thrombosis provides reassurance and aligns with findings from diverse global cohorts [14,16]. Compared with classical APS, which is driven by persistent high-titer antibodies and recurrent thrombosis, COVID-19-related APLAs may be of lower titer, short duration, and limited clinical significance [17].

Although our cohort demonstrated APLA positivity in 30% of patients, the incidence of overt thrombotic events was unexpectedly low (2%). Several factors may explain this discrepancy. First, all patients received at least prophylactic anticoagulation according to national guidelines, which may have substantially lowered the incidence of clinically manifest venous or arterial thrombosis. Second, diagnostic imaging for VTE was not performed routinely and was restricted to cases with clear clinical suspicion due to infection-control limitations during the pandemic. As a result, asymptomatic or subclinical thrombotic events may have remained undetected. Third, early hospitalization and standardized supportive care may have mitigated thrombotic risk compared with overwhelmed health systems worldwide. 

This study has several limitations. First, the absence of a COVID-19-negative or critically ill control group prevents definitive attribution of APLA induction to SARS-CoV-2 rather than to severe systemic inflammation more broadly. Second, the cohort consisted exclusively of patients with severe COVID-19, limiting the ability to explore severity-dependent differences in APLA production. Third, although ELISA-based assays are clinically validated and widely used in APS diagnostics, they lack the analytical depth of multiplex immunoassays or domain-specific antigenic profiling. Lupus anticoagulant testing, frequently the most sensitive marker in acute COVID-19 coagulopathy, was not performed, and we measured only IgG and IgM isotypes of anticardiolipin and anti-β2GPI antibodies, without evaluating IgA isotypes or anti-phosphatidylserine/prothrombin antibodies. Fourth, the 12-week follow-up cohort was modest and susceptible to selection bias, and extended longitudinal assessment was not feasible due to pandemic-related logistical constraints. Fifth, thrombotic events were assessed clinically rather than through routine imaging, which may have led to under-ascertainment of asymptomatic or microvascular thrombosis; histopathological confirmation was similarly not feasible due to ethical and clinical concerns in severely ill patients. Finally, all patients received at least prophylactic anticoagulation, which may have reduced the observed incidence of overt thrombosis but remained ethically required in the management of severe COVID-19.

## Conclusions

In this cohort of patients with severe COVID-19, APLAs were detected in nearly one-third of cases, yet their overall pattern suggested a transient, inflammation-related response rather than markers indicative of classical APS. Consistent with global evidence, our findings do not support routine APLA testing during the acute phase of COVID-19. The immunological mechanisms triggering these antibodies, as well as their long-term clinical relevance, remain uncertain and require larger, multicenter studies with prolonged follow-up. Importantly, distinguishing short-lived, infection-induced APLAs from persistent, pathogenic antibodies is crucial to avoid overdiagnosis of APS and unnecessary long-term anticoagulation, while still ensuring vigilance for the rare patient who may progress to clinically significant thrombotic complications.
